# Exploring the Diagnostic Complexity of Diabetes Subtypes in Pediatric Obesity: A Case Report of an Adolescent With Prader-Willi Phenotype and Literature Review

**DOI:** 10.7759/cureus.66456

**Published:** 2024-08-08

**Authors:** Anca A Boboc, Mara I Ionescu, Elena Tataranu, Catalin Boboc, Felicia Galos

**Affiliations:** 1 Pediatrics, Carol Davila University of Medicine and Pharmacy, Bucharest, ROU; 2 Pediatrics, Marie Curie Emergency Children’s Hospital, Bucharest, ROU; 3 Physiology II - Neuroscience, Carol Davila University of Medicine and Pharmacy, Bucharest, ROU; 4 Functional Sciences, Carol Davila University of Medicine and Pharmacy, Bucharest, ROU; 5 Paediatrics, Faculty of Medicine and Biological Sciences, Stefan cel Mare University of Suceava, Suceava, ROU

**Keywords:** type 1 diabetes mellitus (t1dm), type 2 diabetes mellitus (t2dm), type 1 diabetes mellitus (t1d), children, obesity, diabetes mellitus, prader-willi syndrome

## Abstract

Obesity among adolescents poses a significant global health concern with profound short- and long-term impact on physical and mental well-being. The intricate relationship between obesity and the onset of diabetes remains ambiguous, particularly in cases where the manifestation may differ from that observed in individuals with uncomplicated obesity. Herein, we present the case of a 14-year-old male adolescent with Prader-Willi phenotype and subsequent obesity, exhibiting symptoms of polyuria and polydipsia over a 10-day period, indicative of potential diabetes mellitus (DM). Laboratory assessments revealed a hemoglobin A1c level of 10%, confirming the suspected diagnosis. Notably, despite the absence of ketosis, elevated C-peptide levels and the presence of slightly positive islet-cell antibodies warranted further investigation. While the presence of antibodies typically aligns with a diagnosis of type 1 DM, recent research has highlighted the occurrence of anti-insulin pancreatic cell antibodies in type 2 DM cases. This article aims to delve into the multifaceted issues surrounding adolescent obesity, atypical presentations of DM with positive antibodies, and the long-term management of patients with genetic syndromes.

## Introduction

Across the globe, the increasing rates of obesity pose significant public health challenges and threaten the adequacy of basic healthcare provision in many nations. Obesity independently elevates the risk of cardiovascular diseases and substantially amplifies morbidity and mortality rates. Over the past decades, healthcare expenses have surged due to obesity-related issues among children and adolescents. Childhood obesity is a universal occurrence affecting individuals from diverse socio-economic backgrounds, regardless of age, gender, or ethnicity [1].

Conversely, diabetes represents a major public health challenge associated with high rates of morbidity, hospitalization, health care service use, and mortality. Over recent decades, there has been a consistent rise in the occurrence of both type 1 (T1) and type 2 (T2) diabetes mellitus (DM) among children and adolescents. According to the most recent data from the SEARCH study published in 2023, the adjusted incidence of T2DM among children and adolescents nearly doubled, from 9.0 to 17.9 cases per 100,000 persons per year, from 2002 to 2017 [2]. On the other side, regarding T1DM onset, despite wide global variation, increasing trends in incidence have been observed in most populations, with an average of 3%-4% per year [3]. Nevertheless, with the increasing obesity rates and ethnic diversity among the general pediatric population, relying solely on phenotypic characteristics to differentiate between these diabetes types is becoming more challenging [4]. The overlap between T1DM and T2DM in obese adolescents has led to discussions about mixed forms of diabetes, primarily centered around terminology. However, this scenario does raise compelling questions regarding T1DM management in the presence of insulin resistance.

With this context in mind, we present a case involving an adolescent with diabetes and obesity subsequently related to a genetic syndrome, namely Prader-Willi phenotype. Before delving into the case, a brief overview of this complex genetic syndrome is warranted. Prader-Willi syndrome (PWS), initially characterized by Prader et al. in 1956, manifests as a multisystemic genetic disorder [5]. The prevalence ranges from 1/20,000 to 1/30,000 births, irrespective of race or gender [6]. This syndrome arises from the absence of paternal gene expression on chromosome 15q11.2-q13, leading to dysregulated development and function of the hypothalamus in the brain [7]. In early infancy, it is marked by severe hypotonia, poor appetite, and feeding difficulties. After that, in early childhood, it progresses to excessive eating and gradual development of morbid obesity, unless food intake is strictly controlled. Motor and language developments are delayed, and all individuals exhibit some level of cognitive impairment. Both males and females experience hypogonadism, characterized by genital hypoplasia, incomplete pubertal development, and, in most cases, infertility [8]. Overall, the clinical manifestations encompass specific psychiatric, neuromuscular, and endocrine features, necessitating genetic confirmation for definitive diagnosis [5].

## Case presentation

Presented herein is the case of a 14-year-old male adolescent exhibiting Prader-Willi phenotype, included in the Romanian Association of Patients with Prader-Willi Syndrome, albeit lacking genetic confirmation. Notably, he has battled obesity since the age of four years alongside sleep apnea syndrome. The patient was admitted to our facility due to a 10-day history of polyuria, polydipsia, and abdominal pain, during which he self-monitored elevated glycemic levels reaching 480 mg/dL.

His family history revealed insulin-treated T2DM in both the patient's mother and maternal grandmother, with the mother's condition neglected during pregnancy. Born as the fifth child to non-consanguineous parents, the patient's birth weight was 4,100 g, with normal Apgar score and slightly delayed neurodevelopmental milestones. Notably, he began gaining weight at three years of age, prompting involvement in endocrinological care at the age of four years due to obesity, mental health concerns, and features suggestive of Prader-Willi phenotype.

Genetic assessment commenced in 2011, initiated with a karyotype analysis, revealing a normal chromosomal composition of 46, XY. Further fluorescence in situ hybridization, methylation-sensitive multiplex ligation-dependent probe amplification, and polymerase chain reaction yielded negative results for known genetic aberrations associated with PWS, Angelman syndrome, or fragile X syndrome.

At seven years of age, the patient was diagnosed with right cryptorchidism, which was successfully managed with gonadotropins. Subsequent hormonal evaluations demonstrated normal pituitary function, gonadotropin levels, and testosterone levels, indicative of pubertal progression, consistent over the past two years.

Clinical examination revealed a friendly demeanor alongside moderate mental retardation. Physical characteristics included a height of 162 cm, weight of 160 kg, and a body mass index (BMI) of 61 kg/m², with predominant central adiposity and bilateral lipomastia. Craniofacial dysmorphism encompassed a narrow forehead, hypotelorism, almond-shaped eyes, posteriorly rotated ears, and brachydactyly. Secondary sexual development was complete, with no other clinically notable abnormalities observed.

Laboratory investigations, detailed in Table 1, confirmed a diagnosis of DM, evidenced by a hemoglobin A1c (HbA1c) value of 10%, hyperglycemia on admission (527 mg/dl), absence of ketosis (pH 7.40, bicarbonate 25.7 mmol/L, anion gap 16.60 mmol/L, osmolarity 285 mOsm/kg), and elevated C-peptide levels. Notably, T1DM-specific antibodies were negative, except for slightly positive islet-cell antibodies (ICAs). Thyroid function and testing for celiac disease returned normal results.

**Table 1:** The patient's laboratory results HbA1c, hemoglobin A1c; anti-IA-2 Ab, anti-islet antigen antibody; GAD Ab, anti-glutamic acid decarboxylase antibody; ICA Ab, islet-cell antibodies; TSH, thyroid-stimulating hormone; ATPO, anti-thyroid peroxidase; anti-TG-Ab IgA, anti-transglutaminase antibody immunoglobulin A; anti-TG-Ab IgG, anti-transglutaminase antibody immunoglobulin G

	Patient’s values	Normal values
Glycemia (mg/dL)	527	
HbA1c (%)	10	4.8–5.9
pH	7.40	7.35–7.45
Bicarbonate (mmol/L)	25.7	22.00–26.00
Anion gap (mmol/L)	16.60	8–18.00
Osmolarity (mOsm/kg)	285	280–295
C-peptide (ng/mL)	6.52	1.1–4.4
Anti-IA-2 Ab (IU/mL)	<10	<10
GAD Ab (IU/mL)	<5	<10
ICA Ab (IU/mL)	3.47	<0.9
TSH (mIU/L)	1.75	0.51–4.30
FT4 (ng/dL)	1.48	0.8–1.8
ATPO (IU/L)	9.26	<26
Anti-TG-Ab IgA (U/mL)	<10	<10
Anti-TG-Ab IgG (U/mL)	<10	<10

Basal-bolus insulin therapy was initiated, comprising three doses of rapid-acting lispro insulin (23 IU - 12 IU - 17 IU) and one dose of long-acting detemir insulin (32 IU), totaling 0.52 IU/kg/day, with adjustments based on blood glucose levels and carbohydrate intake. Nutrition education, tailored to provide 210 g of carbohydrates daily, was instituted following a nutritional consultation.

During the subsequent three-month follow-up, notable improvements were observed in the glycemic profile. Consequently, insulin dosage adjustments were necessary, prompting a transition to an oral antidiabetic medication from the biguanides class with a gradually increased dose up to 2,000 mg/day, in conjunction with insulin for correction. After one year of follow-up, the patient demonstrated satisfactory metabolic control (HbA1c between 6.5% and 7%) but exhibited no improvement in weight. Additionally, the ICA levels remained persistently positive, even increasing to 4.48 IU/mL and 8.58 IU/mL after one and two years, respectively, from the initial onset. These antibodies were performed several times since after COVID-19 pandemic many children presented with elevated autoantibodies of different types that spontaneously normalized. Moreover, COVID-19 pandemic is known to have a putative role in the onset of T1DM [9,10]. However, in the presented patient, both anti-IA-2 antibodies and glutamic acid decarboxylase (GAD) antibodies were negative. The C-peptide levels continued to be elevated, reaching 7.83 IU/mL after one year.

## Discussion

Regarding the presented case, the intricate interplay between diabetes and obesity subsequent to Prader-Willi phenotype in adolescents presents a multifaceted clinical challenge, necessitating a comprehensive understanding of underlying pathophysiological mechanisms and evidence-based management strategies.

Double diabetes is a term used to describe individuals who have a combination of both T1DM and T2DM. In the case of PWS, individuals with this condition may initially present with features of T1DM, such as weight loss, high blood glucose levels, and the need for insulin therapy. However, over time, they may develop insulin resistance and require additional treatments such as oral medications or insulin sensitizers, similar to those used in T2DM. Managing double diabetes can be challenging because it requires addressing both insulin deficiency and insulin resistance. In 1995, Zimmet was the first to describe in a group of patients the features of T2DM together with positive anti-islet antibodies [11]. In a Bulgarian adult cohort, 10% of the patients with T2DM had positive antibodies (e.g., anti-glutamic acid decarboxylase, anti-insulinoma-associated 2, and anti-zinc transporter 8 autoantibodies). These patients also presented with worse glycemic control [12]. In children, studies are even more scarce. In 2010, a large clinical trial named Treatment Options for Type 2 Diabetes in Adolescents and Youth (TODAY) screened 1,206 children with T2DM for GAD-65 and insulin-associated protein 2 autoantibodies and reported that 5.9% of the patients were positive for one antibody and 3.9% for both of them [13]. Evidence of islet autoimmunity causing insulin deficiency may be seen in obese children and adolescents with a clinical diagnosis of T2DM. Patients with autoantibodies as a whole have clinical traits that are vastly different from those of patients without them [14,15].

Due to the detection of these antibodies, the International Society for Pediatric and Adolescent Medicine (ISPAD) suggests that testing for islet autoantibodies, such as GAD, islet antigen-2 (IA-2), zinc transporter 8 (ZnT8), and insulin (IAA), should be performed in adolescents clinically diagnosed with T2DM who have not initiated insulin therapy, where feasible [3]. This testing is recommended due to the high prevalence of islet autoimmunity observed in youth with clinically diagnosed T2DM. Studies show that autoantibodies are found in 10% to 20% of adolescents clinically diagnosed T2DM [14,16-21]. The existence of these antibodies correlates with an elevated probability of swiftly advancing to necessitate insulin treatment, alongside an augmented susceptibility to developing additional autoimmune disorders [17]. Moreover, it is crucial to verify diabetes autoantibody testing in overweight or obese pubertal children who exhibit clinical symptoms akin to those of T1DM, such as weight loss, ketosis, or ketoacidosis. While some of these individuals may initially display features suggestive of T2DM, underlying autoimmune processes could be at play. With effective glycemic management, certain individuals may sustain without insulin therapy for prolonged durations, indicating a hybrid manifestation of both T1DM and T2DM. However, youths experiencing hyperglycemia alongside the presence of islet autoantibodies are most accurately classified as having T1DM [21,22].

Another discussion regarding T2DM, obesity, and adolescence is related to maturity-onset diabetes of the young (MODY) [23]. T2DM often manifests during puberty in young individuals, prominently associated with obesity. With the absence of a definitive diagnostic criteria for T2DM and the escalating prevalence of childhood obesity, distinguishing between T2DM and monogenic diabetes in children and adolescents presents formidable challenges [24]. The widespread prevalence of obesity among young populations further complicates the differentiation process, as individuals with monogenic diabetes, who may also present with obesity, overlap with those diagnosed with T2DM. Recent studies underscore this complexity, indicating that 3% of obese youths initially diagnosed with T2DM harbor pathogenic variants linked to monogenic diabetes [25]. Despite these convoluted findings, certain indicators suggestive of monogenic diabetes can aid in the assessment of young individuals initially suspected of having T2DM, including: a) inconsistent severe obesity observed among affected family members, b) the absence of consistent indicators such as acanthosis nigricans or other metabolic syndrome markers (e.g., hypertension, low HDL-cholesterol) among affected family members, c) the presence of diabetes in one parent or other first-degree relatives of that parent, particularly if any affected family member does not display obesity or other metabolic syndrome markers, and d) atypical fat distribution, such as central adiposity combined with slender or muscular extremities [23].

Classifying DM based solely on clinical features can be challenging. An emerging trend is the presentation of numerous cases where individuals remain noninsulin-dependent despite testing positive for autoantibodies at the time of diagnosis, indicating conditions such as type 1.5 DM, latent autoimmune diabetes (LAD) in youth, slowly progressive T1DM, or youth-onset diabetes of maturity. LAD patients typically experience an older age of disease onset and exhibit well-controlled blood glucose levels without requiring insulin injections initially, but they may progress to insulin dependence over several years [26]. Nowadays, it is also termed “LADc - latent autoimmune diabetes in child.”

Obesity and diabetes in children can pose serious problems for diagnosis and treatment. A higher BMI in individuals with T1DM is linked to increased cardiometabolic risk and a greater likelihood of developing chronic complications compared to lean T1DM patients. Conversely, insulin resistance results in elevated insulin needs, which impede glycemic control and weight management [27]. Managing both obesity and T1DM concurrently indeed presents numerous barriers and challenges. The primary challenge lies in finding a delicate balance between intensified insulin therapy, necessary for glycemic control in T1DM, and achieving or maintaining an optimal weight to manage obesity [28]. Addressing these challenges requires a multidisciplinary approach involving healthcare providers specializing in endocrinology, nutrition, exercise physiology, and psychology. Tailored treatment plans that account for individual needs and preferences are essential for optimizing outcomes in individuals with both obesity and T1DM.

While this case report primarily discusses a patient with a Prader-Willi phenotype, it is essential to consider other genetic syndromes that present with similar clinical features of obesity and diabetes. Bardet-Biedl syndrome is one such disorder, characterized by obesity, polydactyly, retinal dystrophy, renal anomalies, and cognitive impairment. Like PWS, Bardet-Biedl syndrome can lead to significant metabolic complications, including T2DM [29]. Alström syndrome, another genetic condition, also shares overlapping features such as progressive obesity, insulin resistance, and T2DM, in addition to sensorineural hearing loss and cardiomyopathy [30]. Cohen syndrome, which involves obesity, hypotonia, intellectual disability, and distinctive craniofacial features, further exemplifies the spectrum of genetic disorders that can complicate the diagnosis and management of obesity and diabetes in pediatric patients [31]. It is crucial to incorporate these differential diagnoses in clinical evaluations to ensure comprehensive care.

The current case underscores the critical importance of ongoing monitoring in patients with Prader-Willi phenotype, as timely therapeutic interventions can effectively mitigate the onset and progression of associated disorders. Despite the absence of genetic confirmation, the clinical manifestation of the Prader-Willi phenotype, alongside neurological and language delays, cryptorchidism, and endocrine abnormalities including obesity and DM, strongly support the diagnosis of PWS in our patient. These findings underscore the imperative for periodic hormonal assessments, akin to those recommended for genetically confirmed PWS cases.

Caused by a genetical alteration located on chromosome 15q11.2-q13, PWS can be explained by three possible mechanisms: a) deletion of a 5-6 Mb region from the paternal contributed chromosome 15 (65-75% of cases), (b) maternal uniparental disomy (20-30% of cases), and (c) imprinting defects that can be sporadic or inherited (only 1-3% of individuals) [32].

Clinical signs first appear during the prenatal period when fetal movement is diminished, the delivery presentation position is abnormal, and there is a greater need for delivery assistance or cesarean parturition [32]. Children with PWS almost always experience central-origin hypotonia accompanied by faint screams, impaired reflexes, including difficulty in sucking, and lethargy. In 90-100% of cases, motor development, including speaking, is delayed by almost double-time period. Intellectual deficits are often modest, even low-normal/borderline intelligence in 40% of the cases [32,33].

Obesity stands as a major cause of morbidity and mortality among individuals with PWS. The fat tissue has a central disposition both in males and females, but, despite this, visceral adiposity is lower than expected [34]. The etiology of obesity in PWS remains incompletely understood, with proposed mechanisms including insulin resistance, caloric imbalance, and hyperghrelinemia [34,35]. Feeding disturbances in PWS gradually turn from poor feeding to hyperphagia, but weight gain usually precedes the appetite increase [36]. Seven phases were outlined in 2011 by Miller et al.: phase 0, characterized by decreased fetal movements and low birth weight; phase 1a, hypotonia with difficulty in feeding (0-9 months), phase 1b, no difficulty in feeding and growing appropriately according to growth curves (9-25 months); phase 2a, weight gain without an increase in appetite or excessive calories (2.2-4.5 years); phase 2b, weight gain with an increase in appetite (4.5-8 years); phase 3, hyperphagia, rarely feeling full (8 years); and phase 4, appetite is no longer insatiable (adulthood) [36].

As a consequence of obesity and insulin resistance, many studies reported a prevalence varying from 7% to 32.5% of T2DM among individuals with PWS, usually diagnosed at adult age (over 20 years old in most studies) [35,37-40]. Although uncommon for the pre-pubertal period, there are reports of T2DM in children with PWS [41]. Obesity and Homeostatic Model Assessment for Insulin Resistance (HOMA-IR) are predictive factors for the development of T2DM, with cut-off values depending on race and ethnicity [41]. Growth hormone therapy and genotype do not influence the development of altered glucose metabolism [34]. Regular monitoring of glycemic metabolism parameters is recommended for early diagnosis and complication prevention [41]. Treatment usually consists of oral hypoglycemic medication, but insulin therapy may be required [42]. Recent case reports suggest a favorable evolution under glucagon-like peptide 1 (GLP-1) analog [34]. If we refer to the presence of positive T1DM antibodies, two cases of T1DM in patients with PWS were reported in the literature, but no correlation can be made [40,41]. Despite the fact that positive antibodies support the diagnosis of T1DM, several studies identified anti-insulin pancreatic cell antibodies in patients with T2DM [43,44].

Distinguishing between the various types of DM poses a significant challenge, especially in young patients with obesity, and particularly in the context of PWS [32]. While the onset of T2DM among patients with PWS is around 20 years of age [32], factors such as family history, gestational diabetes, prolonged obesity, and insulin resistance may support the diagnosis in the current patient. However, in the presented patient, rapid onset of symptoms, elevated HbA1c levels upon hospital admission, and the presence, persistence, and slightly higher value in evolution of positive islet cell autoantibodies are suggestive of a T1DM diagnosis.

In the current patient, diabetes management was particularly challenging due to the multifaceted nature of the disease. Initial basal-bolus insulin therapy effectively improved glycemic control, but the patient eventually required a combination of insulin and an oral antidiabetic drug. This approach highlights the necessity of personalized treatment plans that address both insulin deficiency and resistance. Despite the insulin therapy and dietary interventions, our patient's glycemic control remained stable, but no significant improvement in weight was observed, emphasizing the difficulty of managing obesity-related diabetes in PWS. The persistent elevation of ICA levels in our patient, along with the absence of other diabetes-related autoantibodies, further complicates the diagnosis and management. Regular monitoring of glycemic metabolism and periodic reassessment of autoantibodies are crucial for early detection and prevention of diabetes-related complications. The complexity of the presented case reflects the broader challenges in diagnosing and managing diabetes in the context of obesity.

Irrespective of diabetes classification, early diagnosis and treatment are imperative in PWS to mitigate complications such as diabetic retinopathy, neuropathy, and cardiovascular disease.

Although the current patient most likely has T2DM, the presented clinical features of T1DM, such as the presence of a single positive ICA antibodies and the need for insulin therapy, complicate the diagnosis. This overlap of clinical features suggests a possible hybrid form of diabetes, sometimes referred to as "double diabetes," where the patient exhibits characteristics of both T1DM and T2DM. The patient's persistent hyperglycemia despite significant insulin resistance, coupled with obesity and elevated ICA levels, underscores the complexity of accurately classifying and managing diabetes in individuals with Prader-Willi-like phenotypes. This diagnostic challenge highlights the necessity for continuous monitoring, a personalized treatment approach, and consideration of both autoimmune and metabolic components in the management strategy. This condition characterized by a combination of T1DM and T2DM features likely results from a complex interplay of factors including obesity, impaired beta-cell function, and autoimmune beta-cell destruction [45].

Management of double diabetes in PWS necessitates a multidisciplinary approach, encompassing interventions such as weight management, insulin sensitizers, behavioral therapy, and counseling. Timely detection and appropriate management of double diabetes in PWS can forestall long-term complications and enhance the quality of life for affected individuals. Ongoing research into the underlying mechanisms of double diabetes in PWS is imperative to refine therapeutic strategies and optimize outcomes for individuals grappling with this intricate condition.

Limitations

This case report has several limitations that should be acknowledged. Firstly, the diagnosis of PWS in the presented patient was based on phenotypic characteristics rather than genetic confirmation. While the clinical presentation strongly suggested PWS, the absence of genetic evidence limits the certainty of this diagnosis. Additionally, the unavailability of advanced genetic testing, such as uniparental disomy and array-comparative genomic hybridization, at the time of evaluation further constrains the diagnostic clarity.

Secondly, the discussion of diabetes subtypes in the context of pediatric obesity was largely based on a single case, which limits the generalizability of the findings. A more comprehensive study involving a larger cohort of patients would provide stronger evidence and allow for broader conclusions.

Addressing these limitations in future research could provide more robust insights into the diagnosis and management of diabetes in adolescents with obesity and Prader-Willi phenotype.

## Conclusions

In conclusion, overweight and obesity are becoming more and more frequent among youth with T1DM, leading to significant health issues such as cardiovascular complications and the concurrent diagnosis of T2DM. Double diabetes is a significant medical issue that requires prompt diagnosis and a comprehensive management approach. The condition can present as a combination of T1DM and T2DM, and it is thought to be caused by several factors such as obesity, impaired beta-cell function, and autoimmune destruction of beta cells. Especially, in cases of PW syndrome, phenotype management of double diabetes needs a multidisciplinary approach, including weight management, insulin sensitizers, behavioral therapy, and counseling. Early detection and appropriate management of double diabetes can prevent long-term complications and improve the quality of life of affected individuals. Double diabetes is not unique to PWS, and it is becoming increasingly prevalent in the general population, especially in young people. It is believed to be due to the global rise in obesity rates, sedentary lifestyles, and poor dietary habits. Ongoing research is needed to better understand the mechanisms underlying double diabetes and to develop more effective therapies for this challenging condition. As such, double diabetes is a major public health concern, as it can lead to significant morbidity and mortality if left untreated.
